# Supplementary material for: Galactose to tagatose isomerization at moderate temperatures with high conversion and productivity
Source: Nat Commun. 2019 Oct 7;10:4548. doi: 10.1038/s41467-019-12497-8 (PMC6779876; doi:10.1038/s41467-019-12497-8)
Supplement: Supplementary file 1 — Supplementary Information [file 41467_2019_12497_MOESM1_ESM.pdf]

Galactose to tagatose isomerization at moderate temperatures with high conversion and productivity

Bober & Nair

Supplementary Table 1. Primers used in this study.

Primer	Description	Sequence
oJRB7	Lp_1261_F	GTAAgtcgacAATTTCAAAACAGCTGCAAAAGTAA
oJRB8	Lp_1261_R	AATGgcatgcGCCCCCGTTCTTACCGAGACGGTAT
oJRB10	Lp_2162_F	GTACacgcgtGCTGCCAATGCTGCCTCAAT
oJRB11	Lp_2162_R	GTACaagcttCTACATGGTTGTTTTTTGACTCGT
oJRB14	Lp_1452_F	GCCAgtcgacAAGAAATGGCTCATTGCCCTTGCTGGT GTC
oJRB15	Lp_1452_R	CTAAgcatgcTTGAACCGTGACTTTAGGTTCGTAAGA CTTCC
oJRB20	Lp_2940_F	GTACacgcgtGCCGAATCTAACACTCGAACCGG
oJRB21	Lp_2940_R	ATGCaagcttTTAATCAGTTGTTTTATGGCGCCGTTG
oJRB34	Lp_1261ovrlp_R	ttcatgatgGCTGCCGCGCGGCACCAGgcatgcGCCCCCGT TCTTACCGAGACGGTAT
oJRB35	Lp_1452ovrlp_R	ttcatgatgGCTGCCGCGCGGCACCAGgcatgcTTGAACCG TGACTTTAGGTTCGTAAG
oJRB82	Lp_3014_F	TTGCCAgtcgacAAAAAACTTGTAAGTACAATCGTAA CT
oJRB83	Lp_3014_R	TGCTAAgcatgcAAGGGCCCAAGCAGCCAT
oJRB132	LsLAIovrlp_F	GCATGAgcatgcCTGGTGCCGCGCGGCAGCctcgagTTA AATACAGAAAATTATGAATTT
oJRB133	LsLAIovrlp_R	GTCTACtctagaTTTAATATTGACGTAAGTCAA
oJRB170	LsLAI_IC1_F	GCATTActagATGTTAAATACAGAAAATTATGAATTT TG
oJRB171	LsLAI_IC1_R	GCATTAaagcttTTAGTGATGATGATGATGATGacgcgtT TTAATATTGACGTAAGTCAAATC
oJRB304	LsLAI_SEC_F	TAGGATgtcgacATGGAACAAAAACTTATTTCTGAAG AAGATCTGtctagaATGTTAAATACAGAAAATTATGA ATTT
oJRB305	LsLAI_SEC_R	TCATTAcacgtgTTAATATTGACGTAAGTCAAATCA
oJRB457	LsLAI_IC2_F	TAAGATgccatggtaTTAAATACAGAAAATTATGAATT TTG
oJRB458	LsLAI _IC2_R	CTATTAaagcttTCAATGATGATGATGATGATGTCTAG ATTTAATATTGACGTAAGTCAA

Supplementary Table 2. Plasmids used in this study.

Plasmid	Description	Reference
pLp_3050Ag85B-E6cwa2	pSIP401 based plasmid containing an oncofetal antigen and Lp_2578 anchor for *L. plantarum* surface display.	[1]
pLp_1261Ag85B-E6	pSIP401 based plasmid containing invasion and Lp_1261 anchor for *L. plantarum* surface display.	[2]
pLp_1452Inv	pSIP401 based plasmid containing invasion and Lp_1452 anchor for *L. plantarum* surface display.	[2]
pSIP411	Lactobacillus inducible plasmid system with broad host SH71 origin.	[3]
pJRB01Q-LSH	pSIP401 based plasmid containing Ls-araA, sppQ, Lp_2578, SP_3050, His_6 tag	This work
pJRB02Q-LSH	pSIP401 based plasmid containing Ls-araA, sppQ, Lp_2162, SP_3050, His_6 tag	This work
pJRB03Q-LSH	pSIP401 based plasmid containing Ls-araA, sppQ, Lp_2940, SP_3050, His_6 tag	This work
pJRB04Q-LSH	pSIP401 based plasmid containing Ls-araA, sppQ, His_6 tag	This work
pJRB05Q-LSH	pSIP401 based plasmid containing Ls-araA, sppQ, Lp_1261, SP_3050, His_6 tag	This work
pJRB06Q-LSH	pSIP401 based plasmid containing Ls-araA, sppQ, Lp_1452, SP_3050, His_6 tag	This work
pJRB08Q-LSH	pSIP401 based plasmid containing Ls-araA, sppQ, Lp_3014, SP_3050, His_6 tag	This work
pJRB09Q-LSH	pSIP401 based plasmid containing Ls-araA, SP_3050, His_6 tag	This work
pJRB14Q-LSH	pSIP411 based plasmid containing Ls-araA, sppQ, His_6 tag	This work

Supplementary Table 3. Strains used in this study.

Strain	Description
E. coli NEB 5α	NEB (Beverly, MA)
L. plantarum WCFS1	NIZO Food Research (Kernhemseweg, Netherlands)
A1	*L. plantarum* containing plasmid pJRB01Q-LSH
A2	*L. plantarum* containing plasmid pJRB02Q-LSH
A3	*L. plantarum* containing plasmid pJRB03Q-LSH
A4	*L. plantarum* containing plasmid pJRB05Q-LSH
A5	*L. plantarum* containing plasmid pJRB06Q-LSH
A6	*L. plantarum* containing plasmid pJRB08Q-LSH
SEC	*L. plantarum* containing plasmid pJRB09Q-LSH
IC1	*L. plantarum* containing plasmid pJRB04Q-LSH
IC2	*L. plantarum* containing plasmid pJRB14Q-LSH
IC2 + PBS	Unmodified strain IC2
IC2 + SDS	Modified strain IC2 permeabilized with 0.01% SDS

Supplementary Table 4. Native *L. plantarum* anchor proteins used for LsLAI surface display.

Strain	Anchor protein	Orientation	Type	Ref.
**A1**	Lp_2578	C-terminal	LPxTG	4,5
**A2**	Lp_2162	C-terminal	LysM	6
**A3**	Lp_2940	C-terminal	LPxTG	7
**A4**	Lp_1261	N-terminal	Lipobox	5,8
**A5**	Lp_1452	N-terminal	Lipobox	2
**A6**	Lp_3014	N-terminal	LysM	2,9

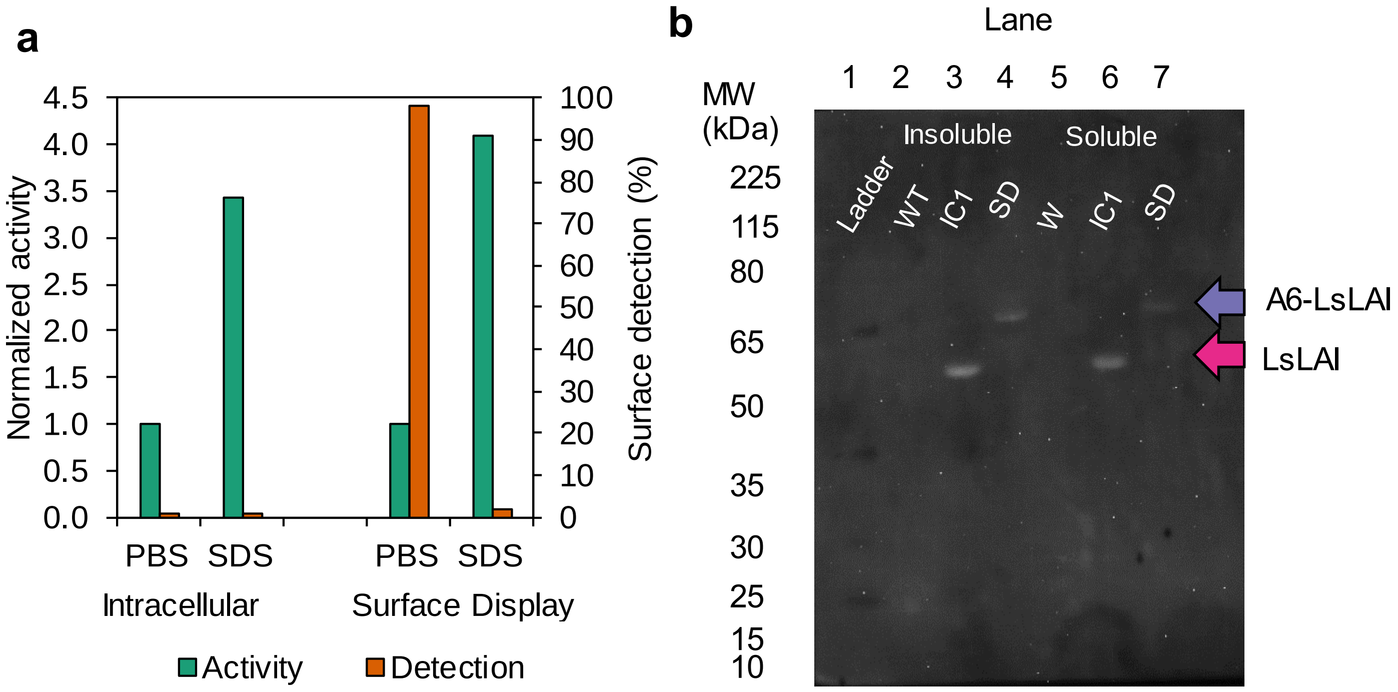

Supplementary Figure 1. Surface treatment of *L. plantarum* surface displayed LsLAI. a) Comparison of the activity (green) and surface detection percentage (orange) of *L. plantarum* expressing LsLAI containing a His_6-tag either intracellularly (left) or surface displayed with anchor protein A6 (right). Activity of cells treated with 0.05 % SDS normalized to untreated cells (PBS). b) Western blot analysis of insoluble (lanes 2 - 4) or soluble protein fraction (lanes 5 - 6) of *L. plantarum* wild-type "WT" (lanes 2, 5) or expressing LsLAI intracellularly "IC1" (lanes 3, 6) or expressing A6-LsLAI surface displayed "SD" (lanes 4, 7). Expected molecular weight (MW) of LsLAI and A6-LsLAI is 54 kDa and 76.5 kDa, respectively. The source data underlying Supplementary Figure 1a are provided in a Source Data file.

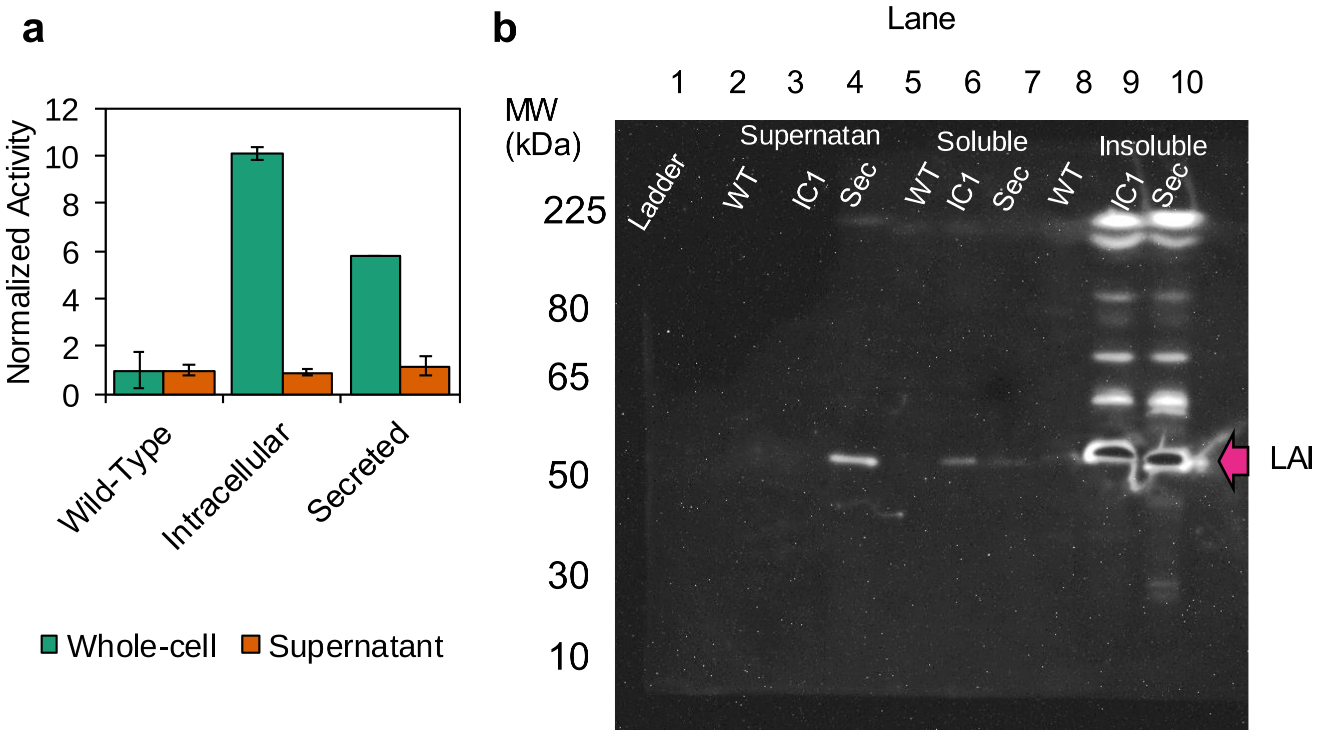

Supplementary Figure 2. *L. plantarum* secreted LsLAI is inactive. a) Comparison of the whole-cell (green) and supernatant (orange) of *L. plantarum* wild-type (left) or expressing LsLAI containing a His_6-tag either intracellularly or as secreted/unanchored protein. The data are means from three biological replicates. b) Western blot analysis of culture supernatant (lanes 2 - 4) or soluble (lanes 5 - 7) and insoluble (lanes 8 - 10) protein fractions of *L. plantarum* cells. Shown are wild-type "WT" control cells (lanes 2, 5, 8), cells expressing LsLAI intracellularly "I" (lanes 3, 6, 9), and cells secreting LsLAI "Sec" (lanes 4, 7, 10). Supernatant was concentrated 20 × before analysis. Expected molecular weight (MW) of LsLAI and secreted LsLAI is 54 kDa. Source data are provided as a Source Data file.

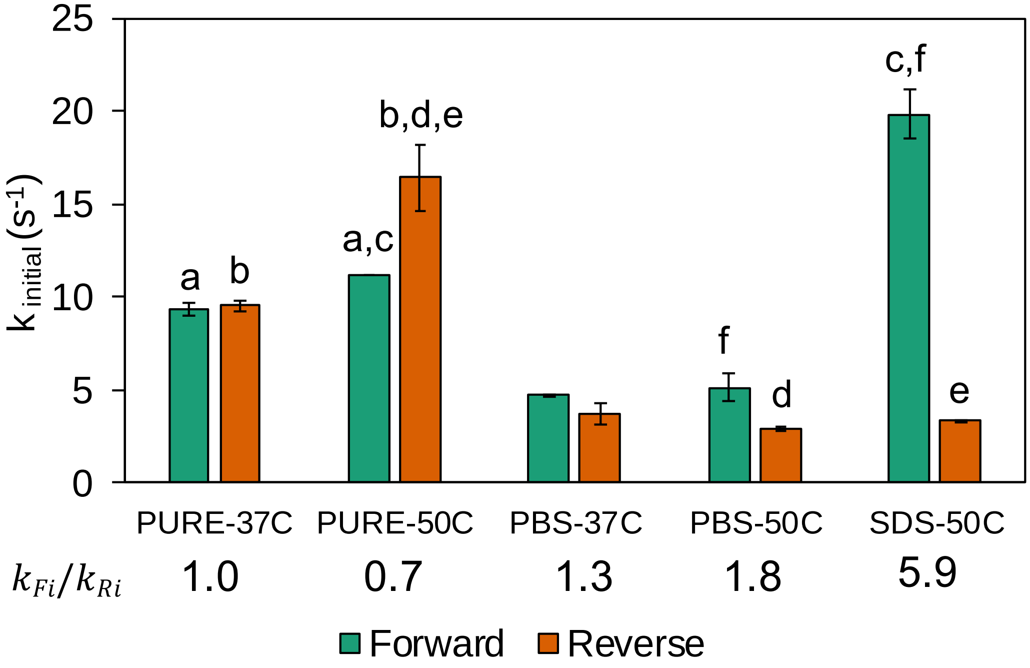

Supplementary Figure 3. Initial turnover rates. Initial turnover rates of purified free-enzyme, (PURE), *L. plantarum*-encapsulated (PBS), and permeabilized *L. plantarum* encapsulated (SDS) LsLAI in forward (galactose as substrate) (green) and reverse (tagatose as substrate) direction (orange) in the presence of 400 mM substrate at 37 or 50 °C. Ratio of forward to reverse reaction rate is denoted by k_{Fi}/k_{Ri}. The data are means from three independent biological replicates (n=3). (Significance between samples tested via ANOVA analysis using SigmaPlot 13.0. a,c,d,e = $p < 0.001$, b = $p < 0.05$) Source data are provided as a Source Data file.

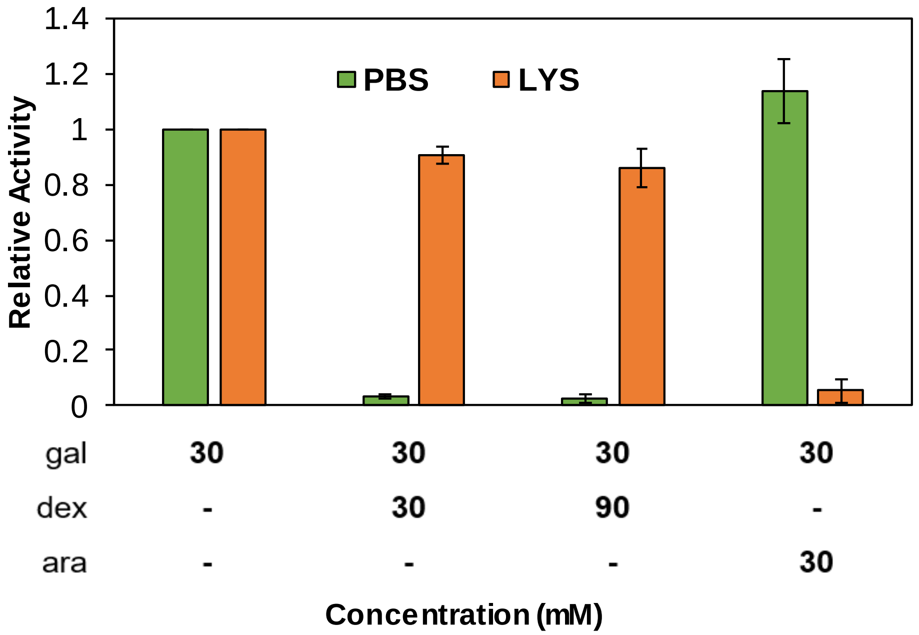

Supplementary Figure 4. Selective nature of cellular encapsulating LsLAI. Tagatose production of unmodified IC2 cells expressing LsLAI (PBS, green) or cell lysate (LYS, orange) in the presence of different combinations of galactose (gal), dextrose (dex), and/or arabinose (ara) after 20 min incubation at 37 °C. Activity normalized to 30 mM galactose condition for unmodified whole-cells (PBS) and cell lysate (LYS) independently. Source data are provided as a Source Data file.

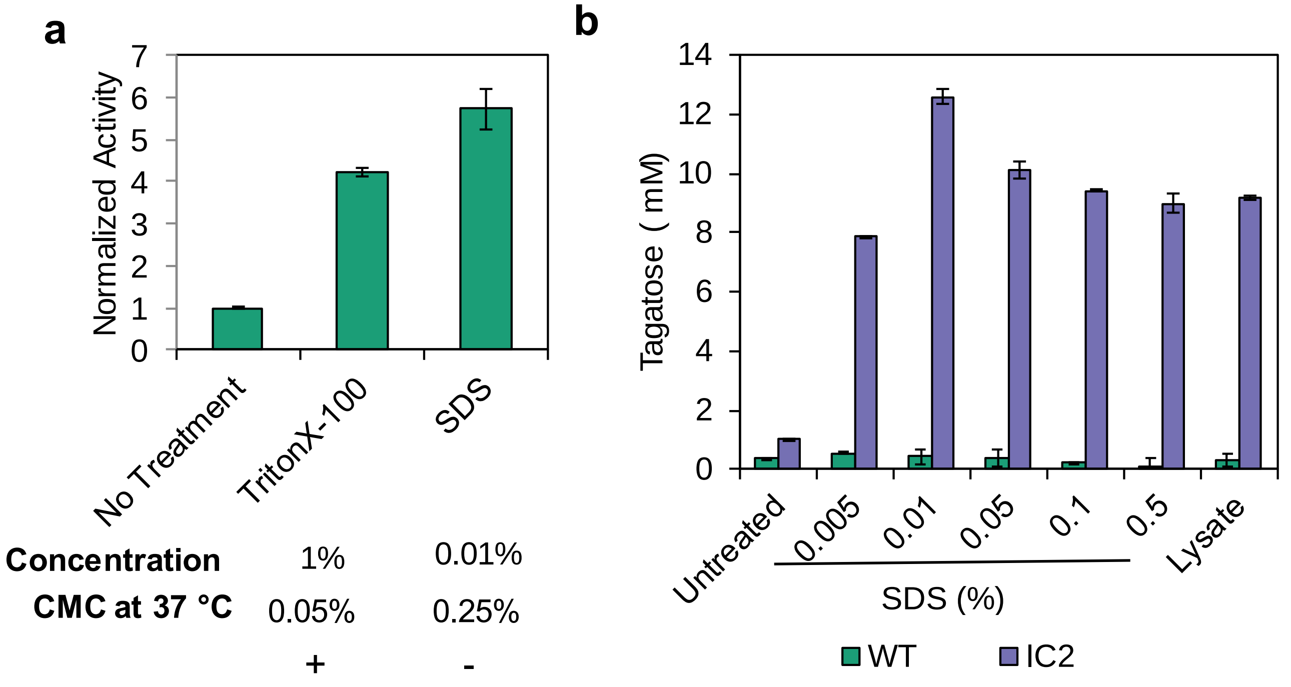

Supplementary Figure 5. SDS permeabilization of encapsulated LsLAI overcomes kinetic penalty. a, Comparison of activity of encapsulated LsLAI having undergone permeabilization by 1 % TritonX-100 (middle) or 0.01 % SDS (right) normalized to untreated cells "No Treatment" (left). b, Optimization of SDS permeabilization of *L. plantarum* wild type "WT" (green) or expressing LsLAI intracellularly "IC2" (purple) that produced the greatest amount of tagatose at 37 °C in 2 h as compared to untreated cells or crude lysate. The data are means from three biological replicates. Source data are provided as a Source Data file.

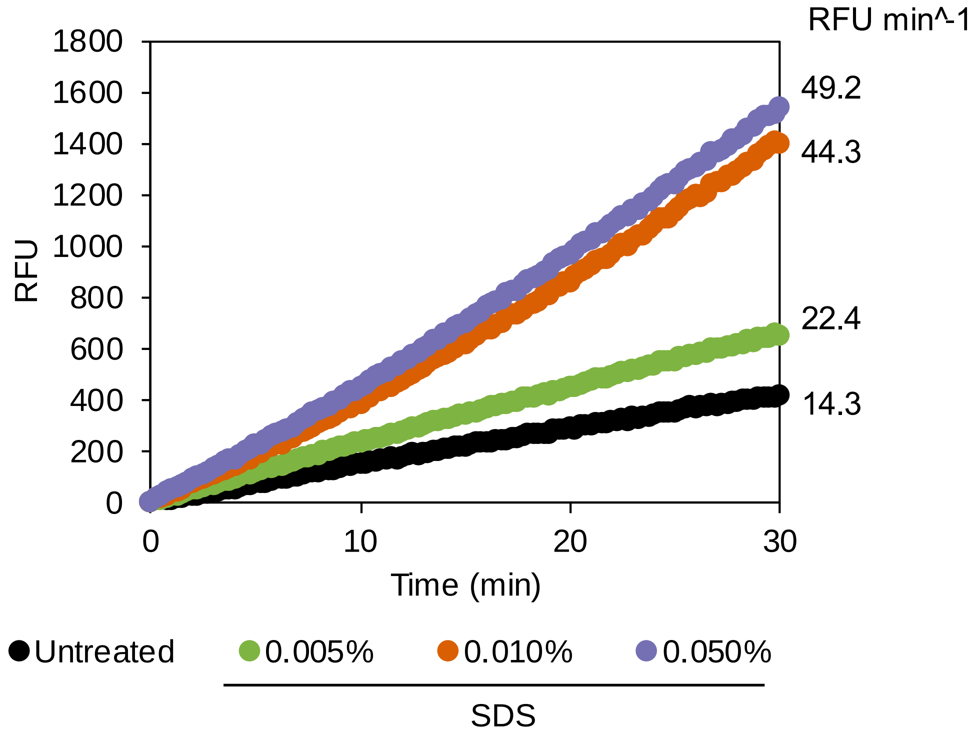

Supplementary Figure 6. SDS permeabilization increases transport. Continuously monitoring the production of fluorescent signal from reporter cFDA after activation upon transport using *L. plantarum* wild-type untreated (black) or after SDS permeabilization with 0.005 % (green), 0.01 % (orange), or 0.05 % (purple) SDS. Relative fluorescence units (RFU) generated per minute is a proxy for transport kinetics. Source data are provided as a Source Data file.

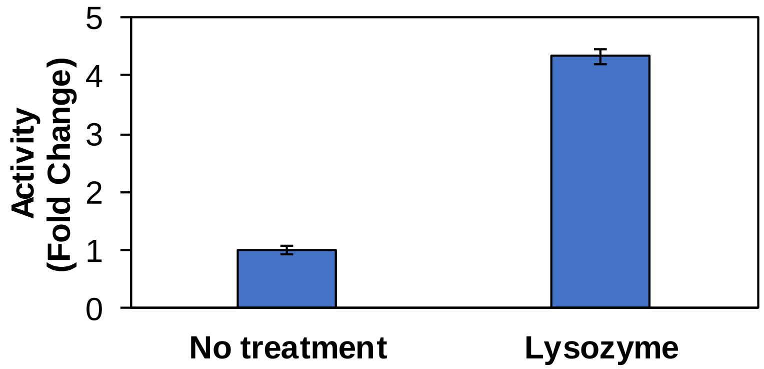

Supplementary Figure 7. Lysozyme treated cells have enhanced tagatose production. Comparing tagatose production of encapsulated LsLAI (IC2) having undergone treatment with 0.01 μM lysozyme (right) normalized to untreated cells “No Treatment” (left) in the presence of 200 mM galactose after 2 h incubation at 37 °C. The data are means from three biological replicates. Source data are provided as a Source Data file.

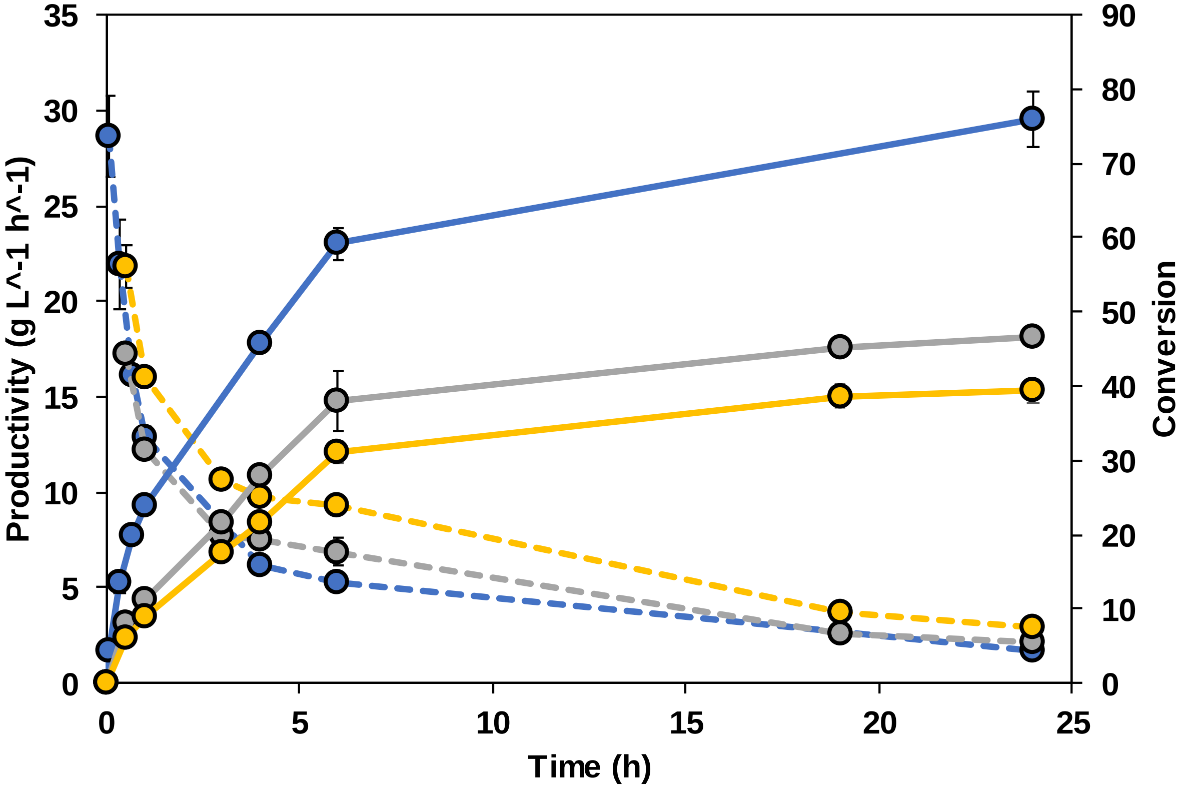

Supplementary Figure 8. Comparing conversion and productivity of SDS treated encapsulated LsLAI at different initial galactose concentrations. Measuring the conversion and average productivity of tagatose production using permeabilized encapsulated LsLAI incubated at 50 °C "SDS-50C" at 300 mM galactose (blue), 600 mM galactose (silver), or 1 M galactose (yellow). Average productivity calculated at each sample timepoint. Inset shows initial reaction rates. Data for 300 mM galactose taken from Figure 4 of this work. The data are means from three biological replicates. Source data are provided as a Source Data file.

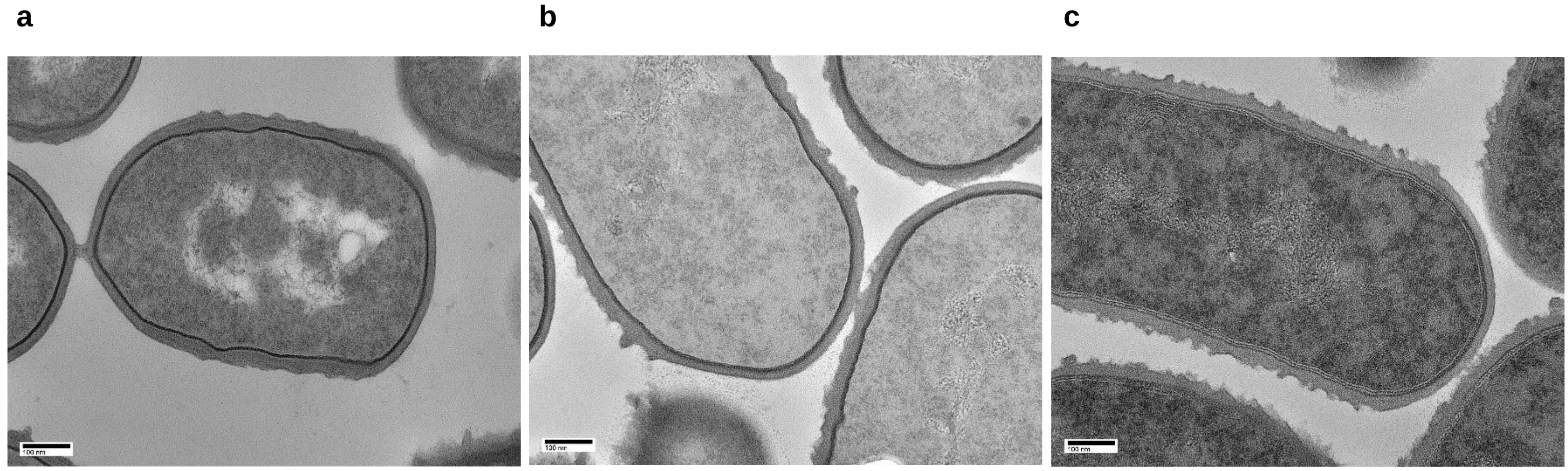

Supplementary Figure 9. TEM analysis of SDS permeabilized *L. plantarum*. Transmission electron microscopy of *L. plantarum* cells to study the effects of 0.01 %SDS permeabilization on cellular structure. a, Wild-type *L. plantarum* in PBS. b, Strain IC2 intracellularly expressing LsLAI in PBS. c, Strain IC2 intracellularly expressing LsLAI treated with 0.01% SDS. HV = 80.0 kV. Direct Mag: 49000×.

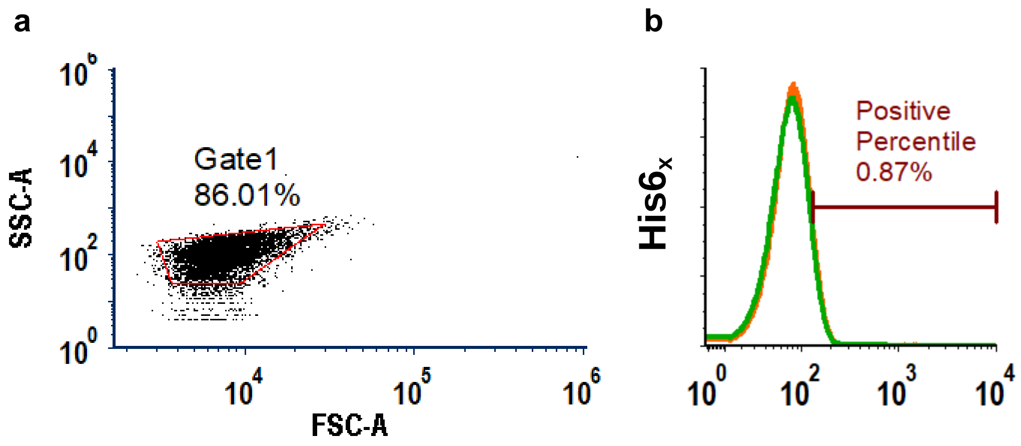

Supplementary Figure 10. Flow cytometry analysis gating. a) Population gating of the negative control (wild-type). Axes are bio-exponential. b) Marker of the negative controls wild-type (green) and intracellularly expressed LsLAI (orange) with His_6-tag. Histogram counts normalized. Plotted on bio-exponential x-axis. Positive percentile marker is shown. Gating strategy used for all flow-cytometry experiments.

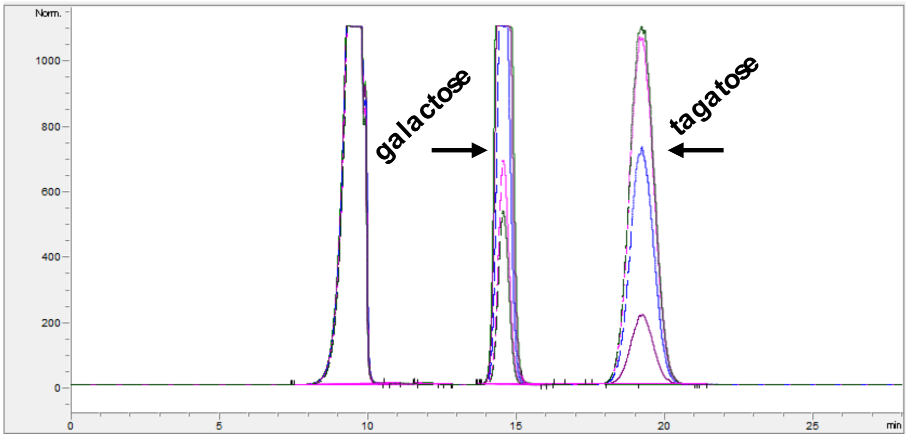

Supplementary Figure 11. HPLC chromatograms of tagatose production samples using strain IC2 + SDS at 50 °C. ELSD chromatogram signals at 0, 6, 24, and 48 h timepoints. One representative figure of triplicate samples.

Supplementary References

1. Fredriksen, L., Mathiesen, G., Sioud, M. & Eijsink, V. G. H. Cell wall anchoring of the 37-kilodalton oncofetal antigen by *Lactobacillus plantarum* for mucosal cancer vaccine delivery. *Appl. Environ. Microbiol.* **76**, 7359–62 (2010).

2. Fredriksen, L. *et al.* Surface display of N-terminally anchored invasin by *Lactobacillus plantarum* activates NF-κB in monocytes. *Appl. Environ. Microbiol.* **78**, 5864–71 (2012).

3. Karlskås, I. L. *et al.* Heterologous protein secretion in lactobacilli with modified pSIP vectors. *PLoS One* **9**, e91125 (2014).

4. Pontes, D. *et al.* Immune response elicited by DNA vaccination using *Lactococcus lactis* is modified by the production of surface exposed pathogenic protein. *PLoS One* **9**, e84509 (2014).

5. Kuczkowska, K. *et al.* Immunogenic properties of *Lactobacillus plantarum* producing surface-displayed *Mycobacterium tuberculosis* antigens. *Appl. Environ. Microbiol.* **83**, e02782-16 (2017).

6. Xu, W. *et al.* Novel surface display system for heterogonous proteins on *Lactobacillus plantarum*. *Lett. Appl. Microbiol.* **53**, 641–648 (2011).

7. Cortes-Perez, N. G. *et al.* Cell-surface display of E7 antigen from human papillomavirus type-16 in *Lactococcus lactis* and in *Lactobacillus plantarum* using a new cell-wall anchor from lactobacilli. *J. Drug Target.* **13**, 89–98 (2005).

8. Nguyen, H.-M. *et al.* Display of a β-mannanase and a chitosanase on the cell surface of *Lactobacillus plantarum* towards the development of whole-cell biocatalysts. *Microb. Cell Fact.* **15**, 169 (2016).

9. Kuczkowska, K., Mathiesen, G., Eijsink, V. G. H. & Øynebråten, I. *Lactobacillus plantarum* displaying CCL3 chemokine in fusion with HIV-1 Gag derived antigen causes increased recruitment of T cells. *Microb. Cell Fact.* **14**, 1 (2015).
