# Supplementary material for: Galactose to tagatose isomerization at moderate temperatures with high conversion and productivity
Source: Nat Commun. 2019 Oct 7;10:4548. doi: 10.1038/s41467-019-12497-8 (PMC6779876; doi:10.1038/s41467-019-12497-8)
Supplement: Supplementary file 3 — Reporting Summary [file 41467_2019_12497_MOESM3_ESM.pdf]

nature research

Corresponding author(s): Nikhil U. Nair

Last updated by author(s): Sep 10, 2019

Reporting Summary

Nature Research wishes to improve the reproducibility of the work that we publish. This form provides structure for consistency and transparency in reporting. For further information on Nature Research policies, see Authors & Referees and the Editorial Policy Checklist .

Statistics

For all statistical analyses, confirm that the following items are present in the figure legend, table legend, main text, or Methods section.

n/a	Confirmed	
☐	☒	The exact sample size (n) for each experimental group/condition, given as a discrete number and unit of measurement
☐	☒	A statement on whether measurements were taken from distinct samples or whether the same sample was measured repeatedly
☐	☒	The statistical test(s) used AND whether they are one- or two-sided<br>*Only common tests should be described solely by name; describe more complex techniques in the Methods section.*
☐	☒	A description of all covariates tested
☐	☒	A description of any assumptions or corrections, such as tests of normality and adjustment for multiple comparisons
☐	☒	A full description of the statistical parameters including central tendency (e.g. means) or other basic estimates (e.g. regression coefficient) AND variation (e.g. standard deviation) or associated estimates of uncertainty (e.g. confidence intervals)
☐	☒	For null hypothesis testing, the test statistic (e.g. F, t, r) with confidence intervals, effect sizes, degrees of freedom and P value noted<br>*Give P values as exact values whenever suitable.*
☒	☐	For Bayesian analysis, information on the choice of priors and Markov chain Monte Carlo settings
☒	☐	For hierarchical and complex designs, identification of the appropriate level for tests and full reporting of outcomes
☒	☐	Estimates of effect sizes (e.g. Cohen's d, Pearson's r), indicating how they were calculated

Our web collection on statistics for biologists contains articles on many of the points above.

Software and code

Policy information about availability of computer code

Data collection	HPLC data were collected using Agilent OpenLab ChemStation Edition C.01.06. Cell density and fluorescence measurements were collected using Molecular Devices SoftMax Pro 6.3. Flow cytometry data collected using
Data analysis	Data analysis was done using Microsoft Excel and SigmaPlot 14.0. Statistical analyses were conducted using SigmaPlot 14.0.

For manuscripts utilizing custom algorithms or software that are central to the research but not yet described in published literature, software must be made available to editors/reviewers. We strongly encourage code deposition in a community repository (e.g. GitHub). See the Nature Research guidelines for submitting code & software for further information.

Data

Policy information about availability of data

All manuscripts must include a data availability statement. This statement should provide the following information, where applicable:

- Accession codes, unique identifiers, or web links for publicly available datasets
- A list of figures that have associated raw data
- A description of any restrictions on data availability

The data that support the plots within this paper and other findings of this study are available from the corresponding author upon reasonable request.

Field-specific reporting

Please select the one below that is the best fit for your research. If you are not sure, read the appropriate sections before making your selection.

☒ Life sciences ☐ Behavioural & social sciences ☐ Ecological, evolutionary & environmental sciences

Life sciences study design

All studies must disclose on these points even when the disclosure is negative.

Sample size	Sample sizes (n=3 biological replicates) were designed to allow verification of in vitro measurements and based on standard practice for similar experiments reported in the literature.
Data exclusions	No data were excluded except where there was clear evidence of an error in the conduct of the experiment or measurement.
Replication	All attempts at replication were successful.
Randomization	Random single genetic isolates of bacterial strains were selected at the start of each experiment.
Blinding	This is not relevant to our study since all experiments were in vitro.

Reporting for specific materials, systems and methods

We require information from authors about some types of materials, experimental systems and methods used in many studies. Here, indicate whether each material, system or method listed is relevant to your study. If you are not sure if a list item applies to your research, read the appropriate section before selecting a response.

Materials & experimental systems

n/a	Involved in the study	
☐	☒	Antibodies
☒	☐	Eukaryotic cell lines
☒	☐	Palaeontology
☒	☐	Animals and other organisms
☒	☐	Human research participants
☒	☐	Clinical data

Methods

n/a	Involved in the study	
☒	☐	ChIP-seq
☐	☒	Flow cytometry
☒	☐	MRI-based neuroimaging

Antibodies

Antibodies used	Alexflour 488 goat anti-mouse (Invitrogen A11001, lot 1890506A), Mouse anti-6X-His (Invitrogen MA1-21315, lot TC261035), Pb pAb to Ms IgG(HRP) (Abcam Ab6728, lot GR198431.2)
Validation	Described in detail on manufactures respective website.

Flow Cytometry

Plots

Confirm that:

☒ The axis labels state the marker and fluorochrome used (e.g. CD4-FITC).

☒ The axis scales are clearly visible. Include numbers along axes only for bottom left plot of group (a 'group' is an analysis of identical markers).

☐ All plots are contour plots with outliers or pseudocolor plots.

☒ A numerical value for number of cells or percentage (with statistics) is provided.

Methodology

Sample preparation	Bacterial cultures were prepared in accordance with manufacturers FC recommendations. Described in Methods Section.
Instrument	Life Technologies Attune NxT, Acoustic Focusing Cytometer, Model: AFC2
Software	De Novo software, FCS Express 6.
Cell population abundance	A total of 10,000 events were collected per sample.

Gating strategy

Initial gating comparing forward-scatter area to side-scatter area was used in order to remove irregular cells from the population. Subsequently a negative gating control for positive results was applied to the labeled wild-type strain.

☒ Tick this box to confirm that a figure exemplifying the gating strategy is provided in the Supplementary Information.
