# Supplementary material for: Galactose to tagatose isomerization at moderate temperatures with high conversion and productivity
Source: Nat Commun. 2019 Oct 7;10:4548. doi: 10.1038/s41467-019-12497-8 (PMC6779876; doi:10.1038/s41467-019-12497-8)
Supplement: Supplementary file 2 — Peer Review [file 41467_2019_12497_MOESM2_ESM.pdf]

Reviewers' comments:

Reviewer #1 (Remarks to the Author):

This manuscript was described on the production of tagatose from galactose with a ratio exceeding the thermodynamic limit of an isomerization of tagatose to galactose using encapsulation and permeabilization of Lactobacillus plantarum expressing L-arabinose isomerase. This manuscript showed novelty on the production of tagatose from lactose with a high ratio of tagatose to galactose and is of interest to readers in the field. However, it was too difficult to understand this manuscript. The manuscript showed too many errors and discussion deviated from the main subject. Some critical methods were skipped. There were some inconsistency between data. Especially, I do not understand the reason that encapsulation shifts the equilibrium toward product formation. Therefore, this manuscript should be rejected.

1. The most critical problem in this manuscript: Authors believed that equilibrium shift toward product formation by encapsulation was due to the substrate (galactose) and product (tagatose) differentially partition across the cell membrane. (Line 155-156) However, the manuscript does not provide strong evidence for its conclusions, the data are not technically sound. The reason why encapsulation differentially partitions substrate and product across the membrane to shift the equilibrium toward product formation was not explained.
Did Lactobacillus plantarum expressing L-arabinose isomerase cause the equilibrium shift toward product formation? Lactobacillus sakei L-arabinose isomerase should be encapsulated and the equilibrium of enzyme encapsulation should be also investigated. When enzyme encapsulation shifted the equilibrium toward product formation, the reason why encapsulation shifted the equilibrium toward product formation should be explained. When enzyme encapsulation did not shift the equilibrium toward product formation, the reason why cell encapsulation shifted the equilibrium toward product formation should be explained. I think that cell encapsulation may affect transporters in cell membrane. Therefore, transporters in cell membrane involved in the partition of substrate and product in the encapsulation should be determined and the equilibrium shift toward product formation should be verified by measuring the rates of galactose and tagatose for encapsulation cells and transporter-knock out encapsulation cells.

2. Authors claimed that the system showed the highest productivity and concentration for food-safe mesophilic tagatose synthesis (Line 23-24, 75-76, and 324-329). However, this is incorrect.
Reference) Guo Q, An Y, Yun J, Yang M, Magocha TA, Zhu J, Xue Y, Qi Y, Hossain Z, Sun W, Qi X “Enhanced D-tagatose production by spore surface-displayed L-arabinose isomerase from isolated Lactobacillus brevis PC16 and biotransformation”. Bioresour. Technol. 247, 940-946 (2018)
According to the reference, spore surface-displayed L-arabinose isomerase produced 100 g/L (553 mM) tagatose from 125 g/L (694 mM) galactose for 28 h, with a productivity of 3.57 g/L/h. The systm in this manuscript produced 45.9 g/L (255 mM) tagatose from 54 g/L (300 mM) galactose for 48 h, with a productivity of 0.96 g/L/h. Therefore, the productivity and concentration of your system were 2.2- and 3.9-fold lower than those of the reported paper, respectively. Please, discuss by comparing your manuscript with the reference.

3. To obtain the higher productivity and concentration of tagatose and to show more exact data, additional data are needed.
1) Tagatose fraction of Fig. 3b at 37 °C was approximately 0.6. However, conversion (tagatose fraction) of Fig. 4 was approximately 0.85 and 0.45 at 50 and 37 °C, respectively. Thus, Tagatose fraction at 37 °C should be measured and presented as Fig. 3c. Please, change Fig. 3c to Fig. 3d.
2) Batch tagatose production were performed from only 300 mM galactose. This galactose concentration was not optimal. To obtain the higher productivity and concentration of tagatose, the experiments by varying the concentration of galactose (e.g. from 200 mM to 1,000 mM) should be performed for 48 h or longer time than 48 h.

3) Permeabilization of E. coli, Gram (−) bacterium, without galactose kinase did cause the equilibrium shift toward product formation because of destruction of cell membrane. However, encapsulation and permeabilization of L. plantarum, Gram (+) bacterium, caused the equilibrium shift toward product formation. Cell membrane structure by permeabilization of Gram (+) bacterium might be different from that by Gram (−) bacterium. Permeabilization of Gram (+) bacterium might cause destroy peptidoglycan layer but not cell membrane. Therefore, cell membrane structure with and without permeabilization of Gram (+) bacterium should be visualized using Transmission Electron Microscopy (TEM).
4) The HPLC results of SDS-50C in Fig. 4 at 0 h, 6 h, and 48 h should be provided in Supplementary data.

4. Spore surface display method in the reference succeeded, whereas your cell surface display method failed. Therefore, all contents for cell surface display method, including text, data in main manuscript, and supplementary data (Line 96-113, Figure 2, part of Table 1, 238-269 Table S1, and Fig. S1) should be removed. Moreover, discussion should be focused on the equilibrium shift toward product formation by encapsulation.

5. Encapsulation is very important in this manuscript. However, this manuscript did not provide encapsulation method. The method for determining turnover rates in Fig. 1 and Fig. S3 was not also provided. Are turnover rates turnover numbers (kcat)?

6. The manuscript showed too many errors and it was difficult to understand this manuscript. Please define more exactly abbreviation.
There was a critical error. I do not know exactly that encapsulation was used for enzyme or cells. In many cases, authors have used enzyme encapsulation like 'encapsulated LsLAI' (line 153). I think that this may be an error because authors have used cell encapsulation.
For examples
1) Line 18: The term of ' at high rates, high conversion, and at high temperatures' should be revised to 'at high rates, high conversion, and high temperatures'.
2) Line 30: The term of 'GRAS (generally regarded as safe)' should be revised to 'generally regarded as safe GRAS)'.
3) Line 103, 108: Please define A1, A2, A3, A4, A5, and A6 with 'Table S1'.
4) Line 110: Please define clearly WT & IC1.
5) Line 110: Please define clearly PBS.
6) Line 124: Please define clearly post-SDS-treatment.
7) Line 166: Please define clearly Strain I.
8) Line 172: The word of 'Permeabilizated' should be revised to 'Permeabilized'.
9) Line 425: Please define clearly IC2.

7. There were inconsistency between data of Fig. 4 and Fig. S3. The conversion by PBS-50C was the almost same as that by SDS-50C in Fig. 4. However, the forward and revers reactions by PBS-50C were distinguishably different as that by SDS-50C in Fig. 4. This is confusing. Please, explain the difference of the data in Fig. 4 and Fig. S3. There were also inconsistency between data of Fig. 3c and Fig. 4. Authors claimed that encapsulation reduced reaction kinetics (Fig. 3c and line 166-171). However, the conversion by encapsulation was higher than purified enzyme (Fig. 4). The exact explanation of the results is required.

8. Please, revise the following contents.
1) The subtitles of Results should be described more detail.
2) Please, transfer Table I to Supplementary data.
3) Legends of Fig. 3c and Fig. S4b: The used concentration of galactose should be presented.
4) All legends should be intensively revised. It was too difficult to understand the legends.

9. In Reference section, there were too many spelling errors and format errors. References should be

revised point by point.
1) Reference numbers 1, 3-17, 19, 20, 22, 23, 26, 27, 32, 34, 35, 37, 38, 46, 50, 54, and 55: The words of 'L-', 'D-',' 'l-', and 'd-' should be revised to the small capital 'L-', and 'D-'.
2) Reference numbers 3, 9, 10, 12, 18, 19, 22, 30, 33, 36, 52, and 53: Titles of papers should be presented as capital for only the first letter in the sentence. For example, the title of "Fermentation of D-Tagatose by Human Intestinal Bacteria and Dairy Lactic Acid Bacteria" in the reference #3 should be revised to Fermentation of D-tagatose by human intestinal bacteria and dairy lactic acid bacteria.
3) Reference numbers 7-11, 13, 15, 16, 21, 23, 25-29, 32, 35, 37-39, 42, 44, 46, 47, 50, 51, 54-56, 58, and 59: The names of microorganisms should be italicized.
The word of 'bacillus licheniformis' in reference #13 should be revised to 'Bacillus licheniformis'.
4) Reference numbers 9, 10, 38, 43, and 52: Doi number should be removed.

Reviewer #2 (Remarks to the Author):

The manuscript report interesting findings on isomerization of galactose into tagatose by whole cell bio-catalysis. The authors argue that the permeabilized cells expressing L-Arabinose Isomerase (LAI) can resolve three (kinetic, thermodynamic, and heat instability) issues of free LAI enzyme-based bioconversions for economic conversion of galactose into tagatose.

The equilibria between galactose and tagatose are dependent on reaction temperatures. As the reaction temperatures increases from 32 to 80 oC, the conversion ratio of tagatose and galactose increases from 32% to 68%. However, enzyme stability will decrease and the Maillard reaction can happen when the reaction temperature is too high. As such, economic production of tagatose from galactose by LAI has been hindered. Using the permeabilized cells with LAI, the authors achieved 85% conversion of galactose into tagatose at 50 C.

While this study reports interesting findings, Kim et al (DOI: 10.1128/AEM.02691-07) already showed the ratio of tagatose and galactose can be shifted substantially from 36% to 68% when whole cells with LAI are used instead of the purified LAI. Although the authors argue that thermo-stability of LAI in the permeabilized L. plantarum can be maintained at 50oC which is higher than the lysis temperature of E. coli, it is not convincing that the presented results might make broader impacts by resolving the persistent issues in sugar isomerization processes.

This reviewer suggests the authors to conduct additional experiments to generalize the idea presented in the manuscript. Fructose isomerization to psicose, another functional sweeter suffers the identical problem. The reaction is catalyzed by D-pscicose 3-epimerase (DPE) but the reaction equilibrium favors the production of fructose. Even though permeabilized cells (not L. plantarum but C. glutamicum) with DPE have been employed, the conversion ratio was only 31% (DOI: 10.1371/journal.pone.0160044). The reviewers and readers will wonder if permeabilized L. plantarum cells with DPE can also increase the rate and ratio of pscicose production from fructose. If yes, the manuscript will report a significant advance in the field and deserve publication in Nature Communications.

Reviewer #3 (Remarks to the Author):

The manuscript examines and attempts to optimize thermodynamic, kinetic and enzyme stability bottlenecks in the production of the industrially important rare sugar tagatose. What is novel is the attempt to address all three bottlenecks together rather than independently as is typically done. Interestingly, when stability and rate improvement was attempted using surface display the author show ample evidence that although the enzyme is displayed, the activity detected is derived from the non-exported cytoplasmic portion of the isomerase and that the surface displayed isomerase was

inactive. This is a significant finding, as almost no studies have examined if the activity in surface display systems is truly derived from the display or from un-exported cytoplasmic protein. Overall, the authors convincingly show that their system produces tagatose in good yields and conversion rates and adds valuable framework to approach future engineering efforts using cell permeabilization as a route to improved production.

Minor comments:

1. There are some minor typos, missing words, and tense issues that require proofreading and correction, but do not inhibit the understanding of the manuscript. Examples: Line 14 should read "…for the non-natural…"; Line 20 should read "…formation, and enables…"; line 71 "used" rather than "use"; line 148 "showed" rather than "shows"; line 187 "has" rather than "has"; line 238 "was" rather than "has", etc.

2. It is stated on line 120 that 0.5% SDS was used to remove surface displayed LsLAI. Should this read 0.05% as is stated in Fig. S1a? Similarly, should 0.001% SDS mentioned on line 199 be 0.01%?

3. Add "SDS" after 0.05% on line 196 for clarity.

4. While discussing the secretion and activity of the LsLAI secreted without an anchor, it was mentioned on line 250 that secretion of proteins is co-translational. While this is true for SRP-dependent secretion of membrane anchored proteins, secretion is post-translational in extracellular or periplasmic proteins that also uses the Sec translocon. Does the LsLAI without an anchor still use the co-translational SRP-dependent pathway?

5. It is stated that there is the highest conversion reported for a mesophilic LAI on line 325-326 and on line 328-329. This is redundant and is suggested that it be edited. Also, there is a period missing between sentences on line 329.

6. Primer sources are mentioned in the methods sections Reagents and Enzymes and Cloning. To avoid redundancy, mention only once.

7. The methods section Cloning is not a description of the cloning. Rather it is of PCR and sequencing. Please rename for accuracy. Similarly, the section Plasmid Construction describes the transformation methods and not plasmid construction. This section should be renamed Plasmid Transformation or Transformation. It should be noted that plasmid construction is not addressed in the methods. This should be addressed.

8. What was the mobile phase used in HPLC? Was it pure water or some other aqueous solution?

9. Figure 2 mentions both the wildtype and his-tagged LsLAI are orange.

10. Table 1 is titled Plasmids and Strains, but no strains are contained in the table. Only plasmids.

Reviewers' comments:

Reviewer #1 (Remarks to the Author):

This manuscript was described on the production of tagatose from galactose with a ratio exceeding the thermodynamic limit of an isomerization of tagatose to galactose using encapsulation and permeabilization of Lactobacillus plantarum expressing L-arabinose isomerase. This manuscript showed novelty on the production of tagatose from lactose with a high ratio of tagatose to galactose and is of interest to readers in the field. However, it was too difficult to understand this manuscript. The manuscript showed too many errors and discussion deviated from the main subject. Some critical methods were skipped. There were some inconsistency between data. Especially, I do not understand the reason that encapsulation shifts the equilibrium toward product formation. Therefore, this manuscript should be rejected.

1. The most critical problem in this manuscript: Authors believed that equilibrium shift toward product formation by encapsulation was due to the substrate (galactose) and product (tagatose) differentially partition across the cell membrane. (Line 155-156) However, the manuscript does not provide strong evidence for its conclusions, the data are not technically sound.

Our response: We thank the reviewer for pointing out the lack of clarity regarding our findings that an equilibrium shift toward tagatose production in *Lactobacillus plantarum* expressing LAI is due to a cell-mediated selective partitioning of sugars. Our data to support this claim is two-fold, 1) initial reaction rate of the cell-encapsulated LAI favored the forward direction (tagatose production from galactose) under all conditions tested while the pure enzyme reaction at 50 °C was faster in the reverse direction (galactose production from tagatose) (Fig. S3) and 2) equilibrium conversion by cell-encapsulated LAI at multiple temperatures (37 °C and 50 °C) and substrate concentrations (30 mM or 300 mM) was higher than that by the pure enzyme system (Fig. 1C vs. Fig. 3B and Fig. 4). This data shows that from both kinetic and thermodynamic perspectives, the cellular encapsulation favors the forward reaction for tagatose production.

The reason why encapsulation differentially partitions substrate and product across the membrane to shift the equilibrium toward product formation was not explained.

Our response: This is an excellent point needed further clarity in the manuscript that “encapsulation” is used to describe cellular encapsulation, i.e. intracellular expression of LAI and not additional chemical encapsulation of cells (e.g. alginate or other polymeric substance). There is precedence in previous literature for this cell mediated effect in *E. coli* where the authors attributed to the selectivity of the galactose transporter MglABC due to a reduction in conversion in the knockout strain.

Did Lactobacillus plantarum expressing L-arabinose isomerase cause the equilibrium shift toward product formation?

Our response: Yes, indeed. The primary results from our work are that *L. plantarum* cells expressing LAI shift the equilibrium of galactose-tagatose isomerization toward tagatose. Fig. 1C vs. Fig. 3B and Fig. 4 also demonstrate a change in the equilibrium.

Lactobacillus sakei L-arabinose isomerase should be encapsulated and the equilibrium of enzyme encapsulation should be also investigated.

Our response: We again apologize for the potentially confusing terminology. As clarified previously, the only encapsulation is within the *L. plantarum* cell. There is so secondary polymeric encapsulation used in the work.

When enzyme encapsulation shifted the equilibrium toward product formation, the reason why encapsulation shifted the equilibrium toward product formation should be explained. When enzyme encapsulation did not shift the equilibrium toward product formation, the reason why cell encapsulation shifted the equilibrium toward product formation should be explained.

Our response: We thank the reviewer for these comments. Our data show that cellular encapsulation of LAI within *L. plantarum* by cytoplasmic expression shifts the equilibrium of the isomerization of galactose to favor tagatose formation under all conditions tested. This is studied relative to free, purified enzyme at both 37 °C and 50 °C and at low (30 mM galactose) and high (300 mM galactose) substrate loadings. We hypothesize that the preferential transport of galactose over tagatose to the cell provides a partitioning that allows the system to circumvent the thermodynamic limitation. To confirm our hypothesis, we show that the forward reaction rate is faster than reverse rate under whole cell conditions, whereas the reverse is true for the purified enzyme (Fig. S3).

I think that cell encapsulation may affect transporters in cell membrane. Therefore, transporters in cell membrane involved in the partition of substrate and product in the encapsulation should be determined and the equilibrium shift toward product formation should be verified by measuring the rates of galactose and tagatose for encapsulation cells and transporter-knock out encapsulation cells.

Our response: Again, there is no secondary encapsulation. But the reviewer makes an excellent point about transport effects. Encapsulation of LAI within the *L. plantarum* cell improved the enzyme's thermal stability and equilibrium conversion. We demonstrated that conversion was improved due to the selectivity of the cellular encapsulation which would be controlled by the relative transport of the two sugars across the cell membrane and wall. In our study, *L. plantarum* was grown on MRS media containing 2% glucose as the carbon source for metabolism. After eight hours of induction in this growth media the cells were washed and added to reaction buffer containing galactose; however, this media lacked other essential components for cellular growth, and we did not observe any galactose metabolism. It is highly likely that the cells are expressing multiple hexose/glucose transporters during the growth and induction phase of the experimental procedure. Therefore, it is unlikely that deletion of any single transporter will be enough to abrogate the substrate partitioning. Further the full repertoire of hexose transporters is not well-described in *L. plantarum*. Therefore, in lieu of a genetic intervention, we undertook a chemical intervention to ascertain the role of transporters. We show that the transport of substrate and product is not based on free diffusion but rather based

on hexose transporters since presence of glucose (a preferred substrate for *L. plantarum*) completely inhibits the LAI reaction in whole cells, whereas arabinose does not (Fig S4). The reverse is true for pure enzyme where arabinose is the preferred substrate over galactose. This demonstrates that in the whole cell system, glucose is an inhibitor of the rate-controlling step (transporter(s)). In the free enzyme system, arabinose is an inhibitor of the rate-controlling step (LAI enzyme itself). Combined these data show that transporter-mediated movement of sugars controls reaction characteristics (and not LAI) in whole cell settings, leading to different equilibrium conversion and kinetic parameters.

We have added the following paragraph to the Results section.

"To further demonstrate that the *L. plantarum* cell acts as a selective barrier, we tested the capacity of other sugars to inhibit the rate controlling step of galactose to tagatose isomerization. Tagatose production by cell encapsulated LAI was significantly inhibited in the presence of glucose while the presence of arabinose had no effect (Fig. S4). Conversely, glucose had minimal inhibition on tagatose production in pure enzyme reactions while arabinose (the preferred substrate for LAI) significantly inhibited the production of tagatose. These data suggest that the rate-controlling step in whole cell system and pure enzyme are completely different – sugar transporters in whole cell systems and passive diffusion in pure enzyme systems. Further, since glucose is a highly preferred substrate of *L. plantarum*, the fact that glucose inhibits whole cell galactose isomerization suggests that the rate-controlling step is one or more glucose-preferring hexose transporter(s)."

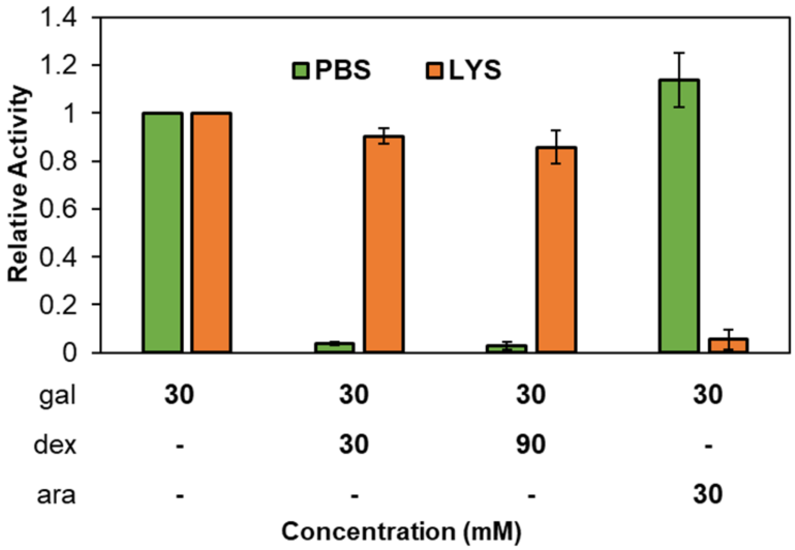

Supplementary Figure 4: Selective nature of cellular encapsulating LsLAI. Tagatose production of unmodified IC2 cells expressing LsLAI (PBS, green) or cell lysate (LYS, orange) in the presence of different combinations of galactose (gal), dextrose (dex), and/or arabinose (ara) after 20 min incubation at 37 °C. Activity normalized to 30 mM galactose condition for unmodified whole-cells (PBS) and cell lysate (LYS) independently. The data are means from three biological replicates.

The method for this experiment was added to the Methods section titled “Enzyme Analysis”.

“The selectivity of the *L. plantarum* cell barrier was tested by comparing the inhibition of the transport of galactose compared to that of the pure enzyme. *L. plantarum* strain IC2 carrying pJRB14Q-LSH was grown and induced for 8 h, as described previously. *L. plantarum* encapsulating LAI (IC2) or purified LAI (PURE) was added to reaction mixture containing and incubated at 37 °C. Reactions proceeded for 20 min before termination as described previously.”

2. Authors claimed that the system showed the highest productivity and concentration for food-safe mesophilic tagatose synthesis (Line 23-24, 75-76, and 324-329). However, this is incorrect.
Reference) Guo Q, An Y, Yun J, Yang M, Magocha TA, Zhu J, Xue Y, Qi Y, Hossain Z, Sun W, Qi X “Enhanced D-tagatose production by spore surface-displayed L-arabinose isomerase from isolated Lactobacillus brevis PC16 and biotransformation”. Bioresour. Technol. 247, 940-946 (2018)
According to the reference, spore surface-displayed L-arabinose isomerase produced 100 g/L (553 mM) tagatose from 125 g/L (694 mM) galactose for 28 h, with a productivity of 3.57 g/L/h. The systm in this manuscript produced 45.9 g/L (255 mM) tagatose from 54 g/L (300 mM) galactose for 48 h, with a productivity of 0.96 g/L/h. Therefore, the productivity and concentration of your system were 2.2- and 3.9-fold lower than those of the reported paper, respectively. Please, discuss by comparing your manuscript with the reference.

Our response: Thank you for bringing this valuable comparison to our attention. Guo et al. reported ~80% conversion from 125 g/L galactose after 28 h and maximum productivity of 4.3 g/L/h at 20 h at 67 °C using their *Bacillus subtilis* spore surface-displayed LAI catalyst in batch process. In their work, they found conversion to be highest (~70%) starting from 50 g/L galactose and decrease as the galactose concentration was increased; reaching only ~25% conversion when starting from 200 g/L galactose (Guo et al Fig. 4a). Productivity peaked at ~2.9 g/L/h when starting with 125 g/L galactose; decreasing both at lower and higher concentrations of galactose. This group improved both conversion and productivity by increasing their catalyst loading (volume of spore suspension) (Guo et al Fig. 4b). With their optimized catalyst and substrate (125 g/L) loading, this group achieved the reported ~80% conversion after 28 h in batch process (Guo et al Fig. 4c).

To address this, we have added Figure S8 showing both the average productivity (g/L/h) and conversion at each of the sampled time points. After 6 h our system achieved a productivity of 9.3 g/L/h, 3 times greater than that reported by Guo and colleagues.

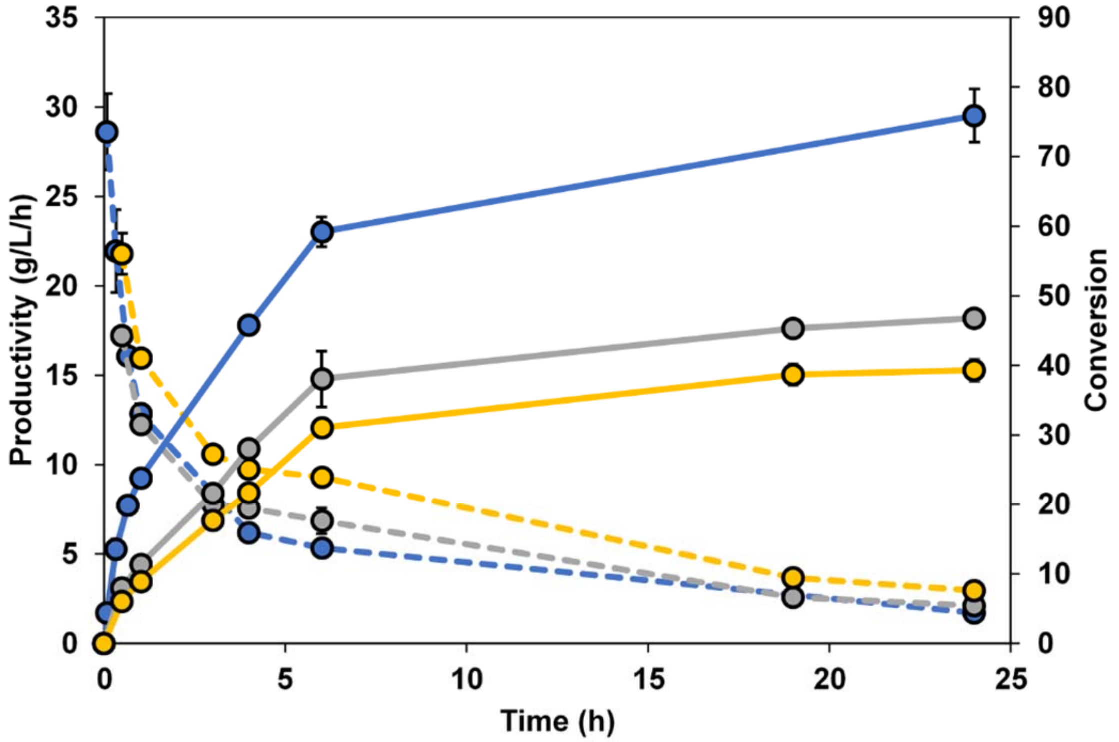

Supplementary Figure 8: Comparing conversion and productivity of SDS treated encapsulated LsLAI at different initial galactose concentrations. Measuring the conversion and average productivity of tagatose production using permeabilized encapsulated LsLAI incubated at 50 °C “SDS-50C” at 300 mM galactose (blue), 600 mM galactose (silver), or 1 M galactose (yellow). Average productivity calculated at each sample timepoint. Inset shows initial reaction rates. Data for 300 mM galactose taken from Figure 4 of this work. The data are means from three biological replicates.

We have added the following text to the Results section.

"To investigate the potential to increase the average productivity of our system, we increased the substrate level from 300 mM to 600 mM and 1 M galactose and measured the production of tagatose using permeabilized *L. plantarum* encapsulating *L. sakei* LAI (strain IC2 + SDS) over the course of 24 h. Higher substrate loading lowered the final conversion of the system to 47 % and 39 % at 600 mM and 1 M galactose respectively (Fig. S8). We observed the highest productivity at low conversions, as expected. After 6 hours, the highest average productivity of our system was 9.3 g/L/h of tagatose starting from 1 M galactose substrate loading compared to 5.3 g/L/h starting from 300 mM galactose. Based on these results we conclude that increasing the substrate loading increases average productivity at the cost of conversion, which is consistent with many previous studies (1-3).”

1. Guo, Q. et al. Bioresour. Technol. 247, 940–946 (2018).
2. Mei, W., Wang, L., Zang, Y., Zheng, Z. & Ouyang, J. BMC Biotechnol. 16, 1–11 (2016).
3. Liu, Y. et al. J. Agric. Food Chem. 62, 6756–62 (2014).

We have added the following text to the Discussion section.

“Average productivity of the modified cell encapsulated LAI batch process could be improved by increasing the initial concentration of galactose from 300 mM to 1 M, albeit with lower conversion.”

We have also adjusted the following sentence to more accurately reflect the productivity comparison to previous findings.

“This is the among the highest reported productivity (averaging ~11.4 over 48 h) for biocatalytic tagatose production, and the highest conversion reported for a mesophilic enzyme (~85 %)”
Has been changed to:
“This is the among the highest reported average productivity (5.3 g/L/h after 6 h) for biocatalytic tagatose production, and the highest conversion reported for a mesophilic enzyme (~85 %)”

3. To obtain the higher productivity and concentration of tagatose and to show more exact data, additional data are needed.
a) Tagatose fraction of Fig. 3b at 37 °C was approximately 0.6. However, conversion (tagatose fraction) of Fig. 4 was approximately 0.85 and 0.45 at 50 and 37 °C, respectively. Thus, Tagatose fraction at 37 °C should be measured and presented as Fig. 3c. Please, change Fig. 3c to Fig. 3d.

Our response: We thank the reviewer for pointing out this difference without further explanation for the results. The difference in conversion obtained from the encapsulated LAI cells is due to the conditions of each batch reaction. In the initial experiment demonstrating the partition effect using encapsulated LAI the galactose concentration used was 10 mM. The system reached a conversion of ~60% after 24 h in batch process at 37 °C, with only ~ 6 mM of tagatose produced. In Fig. 4, the galactose concentration to 300 mM; however, conversion lowered to approximately ~45% at 37 °C. This decline in conversion at high substrate loading is well-documented for this reaction in literature (Guo et al., 2018, Mei et al., 2016, Liu et al., 2014). While the reason for this has not been described, we believe that spontaneous enzyme inactivation after numerous turnovers may contribute to lower final conversion at high substrate loadings.

b) Batch tagatose production were performed from only 300 mM galactose. This galactose concentration was not optimal. To obtain the higher productivity and concentration of tagatose, the experiments by varying the concentration of galactose (e.g. from 200 mM to 1,000 mM) should be performed for 48 h or longer time than 48 h.

Our response: We address this comment in response to your point #2 by increasing the galactose concentration up to 1 M for the reaction to improve the productivity of the system.

c) Permeabilization of E. coli, Gram (−) bacterium, without galactose kinase did cause the equilibrium shift toward product formation because of destruction of cell membrane. However, encapsulation and permeabilization of L. plantarum, Gram (+) bacterium, caused the equilibrium shift toward product formation. Cell membrane structure by permeabilization of Gram (+)

bacterium might be different from that by Gram (−) bacterium. Permeabilization of Gram (+) bacterium might cause destroy peptidoglycan layer but not cell membrane. Therefore, cell membrane structure with and without permeabilization of Gram (+) bacterium should be visualized using Transmission Electron Microscopy (TEM).

Our response: We thank the reviewer for making these points. Kim et al. demonstrated a greater uptake rate for galactose than tagatose in a galactose kinase knockout *E. coli* cell which lead to an increase in tagatose production when expressing LAI. In Fig. S3 we also report an increase in initial galactose isomerization rate compared to tagatose when using *L. plantarum* expressing LAI. Our work demonstrates both the enhanced conversion and increased tagatose productivity in SDS permeabilized cell-encapsulated LAI. Permeabilization increased the reaction rate due to increasing the substrate/product transport across the cellular barrier while maintaining the selectivity.

You suspect that the enhanced transport could be attributed to changes to the Gram-positive cell wall composition. To test this hypothesis, we treated *L. plantarum* encapsulated LAI with lysozyme, an enzyme which catalyzes the destruction of the peptidoglycan component of the gram-positive cell wall (Fig. S7). We observed a similar increase in activity in cells treated with lysozyme to cells chemically treated with SDS detergent compared to untreated cells. These data suggest that the cell wall poses a transport barrier and SDS treatment may be modifying the structure/rigidity of the cell wall to allow faster transport; albeit further direct experimentation should be done to confirm this hypothesis. We thank the reviewer for this keen hypothesis!

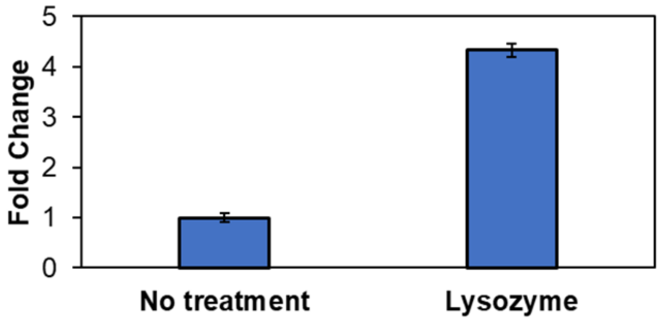

Supplementary Figure 7: Lysozyme treated cells have enhanced tagatose production. Comparing tagatose production of encapsulated LsLAI (IC2) having undergone treatment with 0.01 µM lysozyme (right) normalized to untreated cells “No Treatment” (left) in the presence of 200 mM galactose after 2 h incubation at 37 °C. The data are means from three biological replicates.

We have added the following text to present this finding in the Results section.

“To test whether the enhanced transport could be attributed to changes to the Gram-positive cell wall composition, we mildly treated *L. plantarum* encapsulated LAI (strain IC2) with lysozyme, an enzyme which catalyzes the destruction of the peptidoglycan component of the gram-positive cell wall. These cells displayed a 4.3-fold increase in activity like cells chemically treated with

SDS detergent compared to untreated cells (Fig. S5 and Fig. S7). These data suggest that the cell wall poses a transport barrier and SDS treatment may be modifying the structure/rigidity of the cell wall to allow faster transport; albeit further direct experimentation should be done to confirm this hypothesis. However, transmission electron microscopy (TEM) of SDS-treated *L. plantarum* cells found no evidence of pore formation or leakage of cytoplasmic biological components (Fig. S9)."

We also analyzed SDS-treated *L. plantarum* cells by TEM and found no evidence of pore formation or leakage of cytoplasmic biological components. *L. plantarum* cells treated with 0.01% SDS under the optimized enzyme assay conditions retained their cytoplasmic membranes.

We have added the following TEM images to the Supplemental Information section (Fig. S9) and text to the Discussion section.

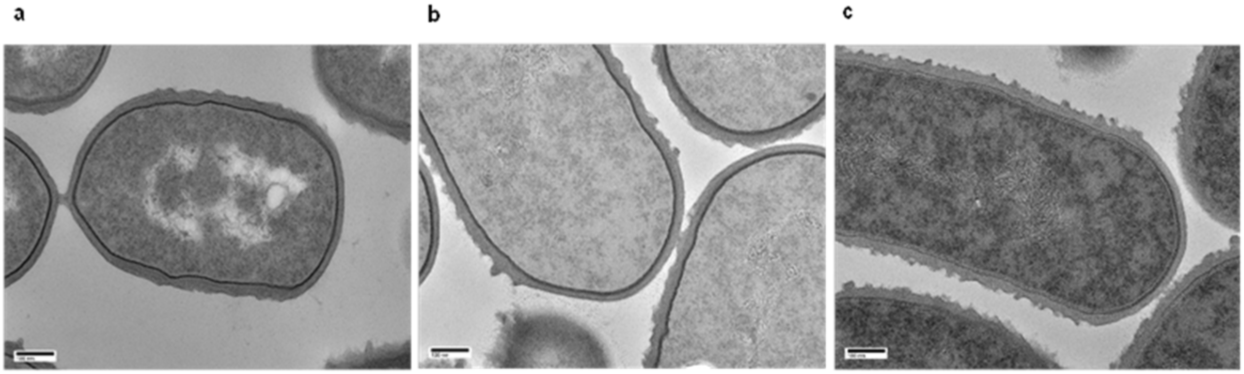

Supplementary Figure 9: TEM analysis of SDS permeabilized *L. plantarum*. Transmission electron microscopy of *L. plantarum* cells to study the effects of 0.01 %SDS permeabilization on cellular structure. a, Wild-type *L. plantarum* in PBS. b, Strain IC2 intracellularly expressing LsLAI in PBS. c, Strain IC2 intracellularly expressing LsLAI treated with 0.01% SDS. HV = 80.0 kV. Direct Mag: 49000×.

"TEM images of SDS-treated and untreated cells further confirmed that the cell wall and membrane were intact under reaction conditions."

d) The HPLC results of SDS-50C in Fig. 4 at 0 h, 6 h, and 48 h should be provided in Supplementary data.

Our response: We have added the HPLC chromatograms for the SDS-50C sample to supplemental information.

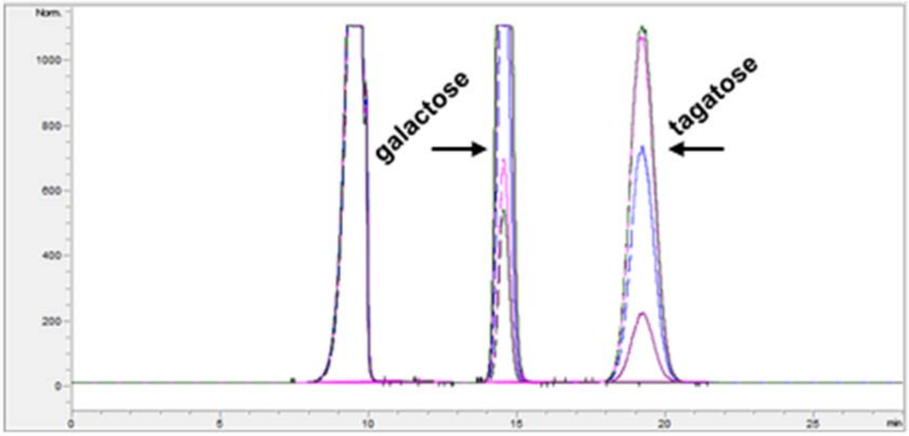

Supplementary Figure 11: HPLC chromatograms of tagatose production samples using strain IC2 + SDS at 50 °C. ELSD chromatogram signals at 0, 6, 24, and 48 h timepoints. One representative figure of triplicate samples.

4. Spore surface display method in the reference succeeded, whereas your cell surface display method failed. Therefore, all contents for cell surface display method, including text, data in main manuscript, and supplementary data (Line 96-113, Figure 2, part of Table 1, 238-269 Table S1, and Fig. S1) should be removed. Moreover, discussion should be focused on the equilibrium shift toward product formation by encapsulation.

Our response: We appreciate the reviewer for this comment. While we were unable to surface display an active LAI on the surface of *L. plantarum*, we provide novel insights regarding the lack of positive correlation between surface detection and surface display activity that will be appreciated by many audiences. Surface display has been shown to improve enzyme stability and therefore it was explored in-depth to improve the activity of LAI at elevated temperatures. Even though the result was negative, it was a key finding that we want to highlight for future lactic acid bacteria surface display applications. Furthermore, Reviewer #3 believes this is a novel and significant finding that would benefit the field.

5. Encapsulation is very important in this manuscript. However, this manuscript did not provide encapsulation method. The method for determining turnover rates in Fig. 1 and Fig. S3 was not also provided. Are turnover rates turnover numbers (kcat)?

Our response: We apologize to the reviewer for the lack of clarity regarding the encapsulation method again. There is no encapsulation other than expressing LAI in *L. plantarum* as an intracellular protein rather than in a lysate or purified sample. The enzyme is localized within the cytoplasm and the whole cell is the catalyst.

In both Fig. 1 and Fig. S3 we have measured the initial reaction rate of each catalyst at a given, non-saturating substrate concentration and stopped the reaction prior to 10 % total conversion. We have updated the methods section to clearly define “$k_{initial}$” and provide further details regarding the execution of this assay.

6. The manuscript showed too many errors and it was difficult to understand this manuscript. Please define more exactly abbreviation.
There was a critical error. I do not know exactly that encapsulation was used for enzyme or cells. In many cases, authors have used enzyme encapsulation like ‘encapsulated LsLAI’ (line 153). I think that this may be an error because authors have used cell encapsulation.
For examples
1) Line 18: The term of ‘ at high rates, high conversion, and at high temperatures’ should be revised to ‘at high rates, high conversion, and high temperatures’.
2) Line 30: The term of ‘GRAS (generally regarded as safe)’ should be revised to ‘generally regarded as safe GRAS)’.
3) Line 103, 108: Please define A1, A2, A3, A4, A5, and A6 with ‘Table S1’.
4) Line 110: Please define clearly WT & IC1.
5) Line 110: Please define clearly PBS.
6) Line 124: Please define clearly post-SDS-treatment.
7) Line 166: Please define clearly Strain I.
8) Line 172: The word of ‘Permeabilizated’ should be revised to ‘Permeabilized’.
9) Line 425: Please define clearly IC2.

Our response: We thank the reviewer for these corrections. We have adjusted the text to define “encapsulation” as “cellular encapsulation through cytoplasmic expression of LAI”. All other corrections were made as suggested.

7. There were inconsistency between data of Fig. 4 and Fig. S3. The conversion by PBS-50C was the almost same as that by SDS-50C in Fig. 4. However, the forward and revers reactions by PBS-50C were distinguishably different as that by SDS-50C in Fig. 4. This is confusing. Please, explain the difference of the data in Fig. 4 and Fig. S3. There were also inconsistency between data of Fig. 3c and Fig. 4. Authors claimed that encapsulation reduced reaction kinetics (Fig. 3c and line 166-171). However, the conversion by encapsulation was higher than purified enzyme (Fig. 4). The exact explanation of the results is required.

Our response: Conversion and reaction rates are largely independent properties. Conversion is a thermodynamic parameter that is determined by the difference in free energy of formation of product and reactant and is independent of the catalyst. Galactose to tagatose conversion/equilibrium is a function of temperature but other reactions may not have a similar positive correlation. Kinetics are a function of the enzyme/catalyst used and are generally positively related to temperature (given by Arrhenius’ law). Under these definitions, rates and conversions are not related – i.e., higher rates do not lead to higher conversion and vice versa. Therefore, we believe that there is no inconsistency in our data.

8. Please, revise the following contents.
a) The subtitles of Results should be described more detail.
b) Please, transfer Table I to Supplementary data.
c) Legends of Fig. 3c and Fig. S4b: The used concentration of galactose should be presented.
d) All legends should be intensively revised. It was too difficult to understand the legends.

Our response: We thank the reviewer for these comments. We have revised the subtitles in results to describe the findings of each section, moved Table 1 to supplementary information,

added the concentration of galactose to Fig. 3c and Fig. S4b, and improved the clarity in all of the figure legends.

9. In Reference section, there were too many spelling errors and format errors. References should be revised point by point.
1) Reference numbers 1, 3-17, 19, 20, 22, 23, 26, 27, 32, 34, 35, 37, 38, 46, 50, 54, and 55: The words of 'L-', 'D-',' 'l-', and 'd-' should be revised to the small capital 'L-', and 'D-'.
2) Reference numbers 3, 9, 10, 12, 18, 19, 22, 30, 33, 36, 52, and 53: Titles of papers should be presented as capital for only the first letter in the sentence. For example, the title of "Fermentation of D-Tagatose by Human Intestinal Bacteria and Dairy Lactic Acid Bacteria" in the reference #3 should be revised to Fermentation of D-tagatose by human intestinal bacteria and dairy lactic acid bacteria.
3) Reference numbers 7-11, 13, 15, 16, 21, 23, 25-29, 32, 35, 37-39, 42, 44, 46, 47, 50, 51, 54-56, 58, and 59: The names of microorganisms should be italicized.
The word of 'bacillus licheniformis' in reference #13 should be revised to 'Bacillus licheniformis'.
4) Reference numbers 9, 10, 38, 43, and 52: Doi number should be removed.

Our response:
We have addressed these issues.

Reviewer #2 (Remarks to the Author):

The manuscript report interesting findings on isomerization of galactose into tagatose by whole cell bio-catalysis. The authors argue that the permeabilized cells expressing L-Arabinose Isomerase (LAI) can resolve three (kinetic, thermodynamic, and heat instability) issues of free LAI enzyme-based bioconversions for economic conversion of galactose into tagatose.

The equilibria between galactose and tagatose are dependent on reaction temperatures. As the reaction temperatures increases from 32 to 80 oC, the conversion ratio of tagatose and galactose increases from 32% to 68%. However, enzyme stability will decrease and the Maillard reaction can happen when the reaction temperature is too high. As such, economic production of tagatose from galactose by LAI has been hindered. Using the permeabilized cells with LAI, the authors achieved 85% conversion of galactose into tagatose at 50 C.

While this study reports interesting findings, Kim et al (DOI: 10.1128/AEM.02691-07) already showed the ratio of tagatose and galactose can be shifted substantially from 36% to 68% when whole cells with LAI are used instead of the purified LAI. Although the authors argue that thermo-stability of LAI in the permeabilized L. plantarum can be maintained at 50oC which is higher than the lysis temperature of E. coli, it is not convincing that the presented results might make broader impacts by resolving the persistent issues in sugar isomerization processes.

This reviewer suggests the authors to conduct additional experiments to generalize the idea presented in the manuscript. Fructose isomerization to psicose, another functional sweeter suffers the identical problem. The reaction is catalyzed by D-pscicose 3-epimerase (DPE) but the reaction equilibrium favors the production of fructose. Even though permeabilized cells (not L. plantarum but C. glutamicum) with DPE have been employed, the conversion ratio was only

31% (DOI: 10.1371/journal.pone.0160044). The reviewers and readers will wonder if permeabilized L. plantarum cells with DPE can also increase the rate and ratio of pscicose production from fructose. If yes, the manuscript will report a significant advance in the field and deserve publication in Nature Communications.

Our response: We thank the reviewer for these comments and the suggestion to demonstrate this strategies ability to improve other reactions suffering from poor thermodynamics, kinetics, and/or enzyme thermal stability. Like the bioproduction of tagatose, production of allulose (formerly referred to as psicose) using the enzyme D-psicose 3-epimerase (DPE) is limited by poor conversion and thermal stability. We cloned the gene encoding DPE from *Clostridium scindens* 35704 into the LAI expression plasmid pJRB14Q-LSH creating pJRB14X-CSFEH and transformed into *L. plantarum* WCFS1. This enzyme was reported to be optimally active at pH 7.5 and 60 °C, have a half-life of 2 h at 50 °C, and achieved a maximum conversion of 28% allulose from fructose at 50 °C (Zhang et al., 2013). We optimized the overexpression of this heterologous enzyme in *L. plantarum* and purified it from the lysate using the encoded C-terminal 6x-His tag using immobilized metal affinity chromatography (IMAC). The activity of the enzyme was measured by the determination of the amount of allulose produced from fructose in a reaction mixture containing 300 mM fructose, 1 mM Mn2+, Tris-HCl buffer (50mM, pH 7.5) and DPE. Samples were taken periodically and the generated allulose was quantified using HPLC running the same method as described for galactose and tagatose separation. Purified *C. scindens* DPE from *L. plantarum* produced allulose at a maximum conversion of 27% (81 mM) and 29% (87 mM) after 33 h in batch process at 37 °C and 50 °C respectively (Figure below); consistent with previous literature (Zhang et al., 2013). Our results suggest that the enzyme is indeed thermally unstable at 50 °C and thus limited in its conversion as seen by the lack of further conversion observed after 6 h. Next we tested if *L. plantarum* encapsulated DPE would improve the reactions conversion through a selective cell-meditated partition of fructose and allulose. *L. plantarum* expressing DPE produced 108 mM and 117 mM allulose from 300 mM fructose after 33 h in batch process corresponding to a maximum conversion of 36% and 38% at 37 °C and 50 °C respectively. The increase in conversion observed using encapsulated LAI was higher than that of the purified enzyme at both 37 °C and 50 °C suggesting that cellular encapsulation overcomes the thermodynamic limitations of the reaction through a cell-mediated partition of fructose and allulose. These results support the potential broad applicability of our reported *L. plantarum* cell encapsulation strategy to produce rare and valuable sugars.

Our results show for the first time, a cell-mediated partition of fructose and allulose in any organism that enhances the maximum conversion obtained by the free enzyme. This is the first report of a DPE being expressed in *L. plantarum* and we report the higher conversion of allulose that reported by Yang et al., 2018. We believe these results to be significant and support the hypothesis that cellular encapsulation improves the thermodynamic limitation of unfavorable rare sugar production; however, our primary focus for the current publication is on the production of tagatose. Furthermore, we believe these DPE results significant enough to warrant an independent manuscript.

1. Zhang, W. *et al. PLoS One* **8**, e62987 (2013).

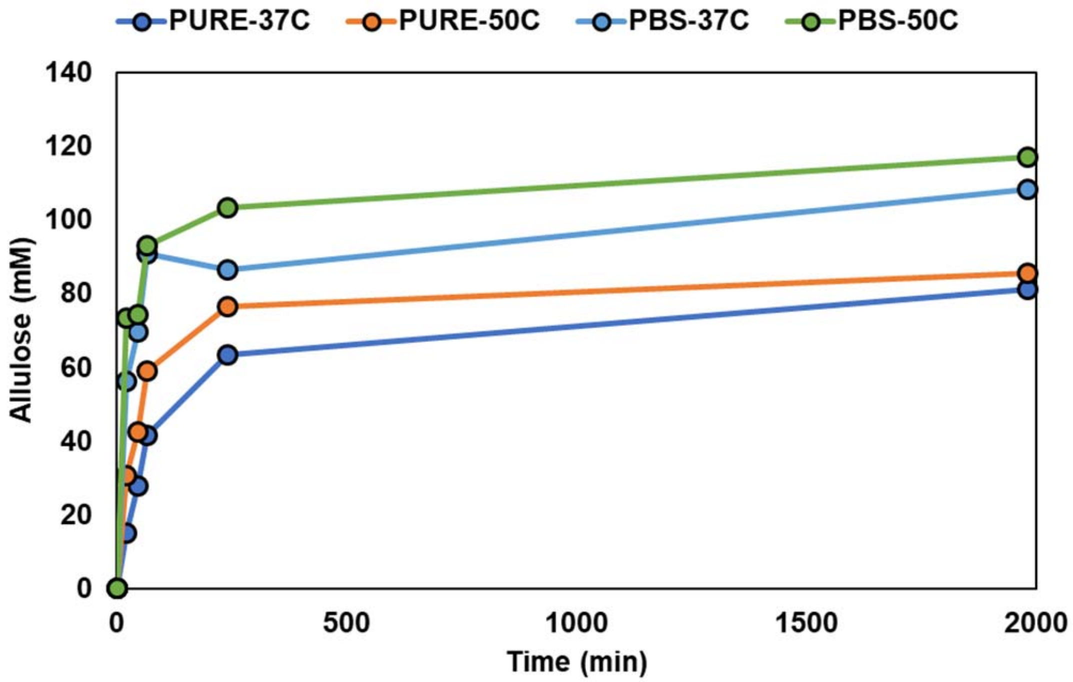

***L. plantarum* cell encapsulated D-Psicose Epimerase also improves allulose production from fructose.** Measuring allulose production using encapsulated CsDPE (PBS) or purified enzyme (PURE) incubated at 37 or 50°C. Greater production of allulose using cellular encapsulated CsDPE compared to purified enzyme.

Reviewer #3 (Remarks to the Author):

The manuscript examines and attempts to optimize thermodynamic, kinetic and enzyme stability bottlenecks in the production of the industrially important rare sugar tagatose. What is novel is the attempt to address all three bottlenecks together rather than independently as is typically done. Interestingly, when stability and rate improvement was attempted using surface display the author show ample evidence that although the enzyme is displayed, the activity detected is derived from the non-exported cytoplasmic portion of the isomerase and that the surface displayed isomerase was inactive. This is a significant finding, as almost no studies have examined if the activity in surface display systems is truly derived from the display or from un-exported cytoplasmic protein. Overall, the authors convincingly show that their system produces tagatose in good yields and conversion rates and adds valuable framework to approach future engineering efforts using cell permeabilization as a route to improved production.

Our response: We thank the reviewer for their kind words.

Minor comments:

1. There are some minor typos, missing words, and tense issues that require proofreading and correction, but do not inhibit the understanding of the manuscript. Examples: Line 14 should

read "…for the non-natural…"; Line 20 should read "…formation, and enables…"; line 71 "used" rather than "use"; line 148 "showed" rather than "shows"; line 187 "has" rather than "has"; line 238 "was" rather than "has", etc.

Our response: Thank you for providing these corrections. All corrections have been made to the manuscript.

2. It is stated on line 120 that 0.5% SDS was used to remove surface displayed LsLAI. Should this read 0.05% as is stated in Fig. S1a? Similarly, should 0.001% SDS mentioned on line 199 be 0.01%?

Our response: We thank the reviewer for pointing this out. A higher concentration of SDS (0.5 %) was necessary to completely remove the surface bound LsLAI for detection in Western-blot analysis (Fig. S1a). Permeabilization of the cell encapsulated LsLAI was optimal at an SDS concentration of 0.01 %. We have fixed the mistake of 0.001 % to 0.01 %.

3. Add "SDS" after 0.05% on line 196 for clarity.

Our response: We have added "SDS" to this location as directed.

4. While discussing the secretion and activity of the LsLAI secreted without an anchor, it was mentioned on line 250 that secretion of proteins is co-translational. While this is true for SRP-dependent secretion of membrane anchored proteins, secretion is post-translational in extracellular or periplasmic proteins that also uses the Sec translocon. Does the LsLAI without an anchor still use the co-translational SRP-dependent pathway?

Our response: We thank the author for bringing this to our attention. We have used the secretion signal component of the membrane protein Lp_3050 as the secretion signal for all surface display and LAI secretion constructs. While it was not confirmed in this study, based on previous reports, we believe this N-terminal signal directs the recruitment of SecA and the protein is co-translationally secreted in an SRP-dependent manner.

5. It is stated that there is the highest conversion reported for a mesophilic LAI on line 325-326 and on line 328-329. This is redundant and is suggested that it be edited. Also, there is a period missing between sentences on line 329.

Our response: We thank the reviewer for identifying this. The redundant sentence has been removed.

6. Primer sources are mentioned in the methods sections Reagents and Enzymes and Cloning. To avoid redundancy, mention only once.

Our response: We have removed the redundant sentence describing the manufacturer of the primers used in this study.

7. The methods section Cloning is not a description of the cloning. Rather it is of PCR and sequencing. Please rename for accuracy. Similarly, the section Plasmid Construction describes

the transformation methods and not plasmid construction. This section should be renamed Plasmid Transformation or Transformation. It should be noted that plasmid construction is not addressed in the methods. This should be addressed.

Our response: We thank the reviewer for this keen observation. We have revised the Methods section to accurately reflect the methods described by combining the previously named “Cloning” and “Plasmid Construction” sections to “Plasmid and Strain Construction”. Additionally, we have added tables for all primers, plasmids, and strains used in this study to supplemental information.

8. What was the mobile phase used in HPLC? Was it pure water or some other aqueous solution?

Our response: We thank the reviewer for pointing out this lack of clarity. The mobile phase was deionized water. We have replaced the sentence reading “Aqueous mobile phase was run...” with “The mobile phase was filtered deionized water run...”

9. Figure 2 mentions both the wildtype and his-tagged LsLAI are orange.

Our response: Wild-type cells were displayed as the orange histogram in Figure 2. We have changed the legend description to this correction.

10. Table 1 is titled Plasmids and Strains, but no strains are contained in the table. Only plasmids.

Our response: Thank you for this observation. Based on the reviewer’s comments we have separated this information into three supplementary tables - Table S1: Primers used in this study; Table S2: Plasmids used in this study; Table S3: Strains used in this study.

REVIEWERS' COMMENTS:

Reviewer #1 (Remarks to the Author):

I am satisfied with Author's response.
I did not understand the term of‟encapsulation" at the previous review. I though that encapsulation was artificial chemical membrane. Your description that‟encapsulation" was used to describe cellular encapsulation made the explanation of equilibrium shift. Thus, the term of all‟encapsulation" in the manuscript should be revised to "cellular encapsulation".
This revised manuscript have high novelty and originality and is now acceptable for publication.

Reviewer #2 (Remarks to the Author):

The authors conducted the additional experiment suggested by this reviewer. While the improvement of allulose production from fructose by was not as drastic as tagatose production from galactose, the newly presented result can strengthen the main argument of the manuscript.

However, the authors did not include the new result (provided in their response) into the revised manuscript. They can add a small paragraph on validation of their concept using the second example into Result or Discussion section.

This reviewer suggests to add the result of allulose production into Main text.

Reviewer #3 (Remarks to the Author):

The revised manuscript addresses all the reviewer’s comments and adds clarity.

Reviewers' comments:

Reviewer #1 (Remarks to the Author):

I am satisfied with Author's response.
I did not understand the term of“encapsulation" at the previous review. I though that encapsulation was artificial chemical membrane. Your description that“encapsulation" was used to describe cellular encapsulation made the explanation of equilibrium shift. Thus, the term of all“encapsulation" in the manuscript should be revised to "cellular encapsulation".
This revised manuscript have high novelty and originality and is now acceptable for publication.

Our response: We thank the reviewer for improving the language used in the manuscript to clearly define cellular encapsulation. We have revised the manuscript to reflect these suggestions.

Reviewer #2 (Remarks to the Author):

The authors conducted the additional experiment suggested by this reviewer. While the improvement of allulose production from fructose by was not as drastic as tagatose production from galactose, the newly presented result can strengthen the main argument of the manuscript.

However, the authors did not include the new result (provided in their response) into the revised manuscript. They can add a small paragraph on validation of their concept using the second example into Result or Discussion section.

This reviewer suggests to add the result of allulose production into Main text.

Our response: We greatly thank the reviewer for their feedback regarding the discovery of this newly presented result. While we agree this result strengthens the argument of the manuscript, we believe the results and discussion necessary to include this data in the Main text to be outside the scope of the present manuscript. We have elected to be transparent in the peer review process thereby displaying the positive results publicly, albeit not in the main text. We have also adjusted the following discussion point in the manuscript to more accurately reflect this result and future work.

The sentence:
“We believe that a similar strategy may be applicable to other biocatalytic systems as well – particularly lyases, transglycosidases, and isomerases – where free enzyme performance is poor due to stability, thermodynamic, and/or kinetic limitations.”

Was replaced with:

We believe that a similar strategy may be applicable to other biocatalytic systems as well – particularly lyases, transglycosidases, isomerases, and epimerases – where free enzyme

performance is poor due to stability, thermodynamic, and/or kinetic limitations.

Reviewer #3 (Remarks to the Author):

The revised manuscript addresses all the reviewer’s comments and adds clarity.

Our response: We thank the reviewer for their feedback and suggestions on the manuscript.
